# Foraging Strategies of Laysan Albatross Inferred from Stable Isotopes: Implications for Association with Fisheries

**DOI:** 10.1371/journal.pone.0133471

**Published:** 2015-07-31

**Authors:** Ann E. Edwards, Shannon M. Fitzgerald, Julia K. Parrish, John L. Klavitter, Marc D. Romano

**Affiliations:** 1 Alaska Fisheries Science Center, National Oceanic and Atmospheric Administration, Seattle, Washington, United States of America; 2 School of Aquatic and Fisheries Sciences, University of Washington, Seattle, Washington, United States of America; 3 Midway Atoll National Wildlife Refuge, United States Fish and Wildlife Service, Papahānaumokuākea Marine National Monument, United States of America; 4 Migratory Birds and Habitat Programs, United States Fish and Wildlife Service, Portland, Oregon, United States of America; Institute of Ecology, GERMANY

## Abstract

Fatal entanglement in fishing gear is the leading cause of population decline for albatross globally, a consequence of attraction to bait and fishery discards of commercial fishing operations. We investigated foraging strategies of Laysan albatross (*Phoebastria immutabilis*), as inferred from nitrogen and carbon isotope values of primary feathers, to determine breeding-related, seasonal, and historic factors that may affect the likelihood of association with Alaskan or Hawaiian longline fisheries. Feather samples were collected from live birds monitored for breeding status and breeding success on Midway Atoll in the northwestern Hawaiian Islands, birds salvaged as fisheries-bycatch, and birds added to museum collections before 1924. During the chick-rearing season (sampled April-May), means and variances of stable isotope values of birds with the highest, most consistent reproductive success were distinct from less productive conspecifics and completely different from birds caught in Hawaiian or Alaskan longline fisheries, suggesting birds with higher multi-annual reproductive success were less likely to associate with these fisheries. Contemporary birds with the highest reproductive success had mean values most similar to historic birds. Values of colony-bound, courting prebreeders were similar to active breeders but distinct from prebreeders caught in Alaskan longline fisheries. During the breeding season, δ^15^N values were highly variable for both contemporary and historic birds. Although some historic birds exhibited extremely low δ^15^N values unmatched by contemporary birds (< 11.2‰), others had values as high as the highest fishery-associated contemporary birds. During the non-breeding season (sampled July-September), isotopic variability coalesced into a more narrow set of values for both contemporary and historic birds. Our results suggest that foraging strategies of Laysan albatross are a complex function of season, breeding status, and multi-annual breeding success, factors that likely affect the probability of association with fisheries.

## Introduction

Fatal entanglement in fishing gear has become a major cause of seabird population decline globally because birds are attracted to fishing operations to feed on bait and fisheries waste [1]. The majority of albatross species are threatened with extinction, and the most significant cause of population decline is association with longline fisheries where albatrosses are hooked after ingesting bait, and drowned as the hook descends to fishing depth [2]. Breeding-related and environmentally-driven changes in foraging opportunities are known to affect spatial and temporal overlap of seabirds with regional fisheries [3–5]. The relative availability, and not necessarily the nutritional quality, of fisheries-associated food can be attractive to seabirds [6]. Rates of association with fisheries vary between individual albatross [7]. Thus, the probability that seabirds associate with fisheries can change, among other things, with change in breeding-associated foraging distribution, availability of natural prey, or individual choice.

Where seabirds forage is shaped by prey availability that can change seasonally [3], [8–10] and annually [11–14]. Where seabirds forage is also affected by their breeding status. Active breeders, which return to the breeding colony to tend mates, nests, eggs, or chicks, forage within a maximum radius from the colony [15] with the radius dependent on the breeding stage-specific frequency of colony attendance [16–17]. Non-breeders, including prebreeders, failed breeders and those that choose to skip a year of breeding, are theoretically unconstrained by a maximum foraging radius relative to the breeding colony [15], a freedom they can exploit to varying degrees [18]. Individual choice, which is measurable as a repeated pattern, also can affect seabird foraging behavior [7], [19–20]. Within a population most albatrosses visit similar mesoscale oceanographic features [14], [21–22], but at the individual level, knowing the location of profitable and predictable foraging zones can provide a fitness advantage [23]. Foraging choices by individuals improve with age and breeding experience, as measured by annual reproduction and survival [24–27]. On average, foraging choice within age or experience cohorts improve via a reduction over time in foraging heterogeneity [24–26].

Albatrosses are long-lived, low-fecund species [28] for which trade-offs in reproductive success occur between years [29–32]. Albatrosses limit annual reproduction to a single, very slow growing chick, an adaptation that has enabled them to exploit food dispersed widely, patchily, and relatively unpredictably across vast oceans [23], [28]. Adult albatrosses buffer themselves against unpredictable food availability by limiting production of large, expensive eggs, abandoning reproductive effort when their body condition falls below a critical threshold [12], [28], [33], and skipping breeding entirely during poor foraging years [12], [16]. Albatrosses can use a year of skipped or failed breeding to boost body condition and hence reproductive success in the subsequent year [29–32]. Consequently for albatross species defined as annual breeders, an individual’s breeding quality is best measured by its fledging success over a minimum of two consecutive years.

Laysan albatross (*Phoebastria immutabilis*) breed predominantly on Midway Atoll, a federally protected National Wildlife Refuge, part of the Papahanaumokuakea Marine National Monument, in the leeward Hawaiian Islands [34–35]. They also breed on much smaller colonies across the Hawaiian Island chain, Wake Island, Japan and Mexico [36]. Laysan albatross forage widely across the North Pacific Ocean and associated northern seas [37] with maximum foraging trip distance during chick rearing ranging from 51 to 4010 km [16]. Although their foraging distribution includes most of the North Pacific, Laysan are concentrated within the mid-to-northern regions of the North Pacific Transition Zone (NPTZ) [14], [21], [37–38]. Less commonly, Laysan albatross are found along the continental shelf and shelf break, predominantly north-to-northwest of the Hawaiian Islands, including into the Bering Sea and Sea of Okhotsk [16], [19], [37–38]. Laysan albatross feed on free-ranging fish, squid, and crustaceans [39–40], as well as fisheries-associated bait, target catch, offal, and discards [41–42]. The division of these food sources within and among demographic groups or seasons is poorly understood.

Laysan albatross are caught incidentally in Hawaiian and Alaskan longline fisheries, and in longline fisheries of the central North Pacific [1]. In Hawaii, Laysan albatross are caught by the shallow-set swordfish (*Xiphias gladius*) and deep-set tuna (*Thunnus* spp.) longline fisheries, which operate near the subtropical gyre boundary [43]. In Alaska, Laysan albatross are most commonly caught along the Aleutian archipelago in the sablefish (*Anoplopoma fimbria*), Greenland turbot (*Reinhardtius hippoglossoides*) and Pacific cod (*Gadus macrocephalus*) longline fisheries [44]. Laysan albatross are also caught in the high seas tuna fisheries of Japan and Taiwan, which operate within core albatross foraging areas of the western North Pacific [1], [45].

Stable isotope values provide a robust tool for quantifying similarities and differences between birds of unknown history salvaged after fatal entanglement in fisheries and birds monitored over time for reproductive success. Stable isotope values of nitrogen (the relative ratio of ^15^N to ^14^N, referred to as δ^15^N) and carbon (the relative ratio of ^13^C to ^12^C, referred to as δ^13^C), which are often correlated with each other [46], have been used widely to provide indices of assimilated diet and to estimate species-specific or guild-specific foraging niche widths [46–51]. Additionally, documentation of geographic gradients of δ^15^N and δ^13^C within marine environments [52–54] has enabled inferences to be made from nitrogen and carbon isotope values about foraging locations for seabirds that span marine realms [19], [44], [55–59]. Thus for far-ranging seabirds, such as Laysan albatross, “foraging strategy”, as inferred from δ^15^N and δ^13^C, is a composite term that encompasses both the foraging location and the trophic level of the prey consumed.

We investigated foraging strategies of Laysan albatross, as inferred from δ^15^N and δ^13^C, to assess breeding-associated, seasonal, and historic factors that may affect the likelihood of association with Alaskan or Hawaiian longline fisheries. We compared isotope values among sampling categories that included breeding season (chick-rearing in April-May, or non-breeding in July-September), breeding status (active breeder, failed breeder or courting prebreeder), and breeding quality (one versus two chicks fledged over consecutive breeding seasons). For each season, we compared stable isotope values from each breeding category to values of two out-classes: birds salvaged from longline fishing operations in Alaska or Hawaii, and historic birds (museum specimens) that lived before the advent of industrial fishing. We used our results to infer relationships between breeding status/quality/season and foraging strategy, especially in relation to the likelihood of association with commercial fisheries.

## Methods

Samples of flight feathers from live Laysan albatross of known breeding status and breeding quality (number of chicks fledged in two consecutive years) were collected with permission (United States Fish and Wildlife migratory bird collection permit MB136837-1, and Papahanaumokuakea Marine National Monument collection permit NWHIMNM-2007-007) on the breeding colony (Sand Island National Wildlife Refuge, Midway Atoll, 28.20°N, 177.35°W). Samples of primary feathers were taken from dead birds collected by fisheries observers in the Hawaiian pelagic longline fisheries for swordfish and tuna (USFWS migratory bird collection permit MB035470-0), and also in the Alaskan groundfish longline fisheries (USFWS migratory bird collection permit MB052060-0). Samples of flight feathers from birds that were alive before the advent of large-scale fishing in the North Pacific were sampled from specimens collected before 1924 curated at the National Museum of Natural History, and at the Burke Museum at the University of Washington.

Feathers are metabolically inert enabling comparisons in stable isotope values between recent and historic samples [60]. However, fossil fuel and forest burning over the last century have caused a systematic increase in dissolved carbon in the marine system resulting in a measurable increase in δ^13^C values over time, a process known as the “Suess Effect” [53]. A calibration equation can facilitate comparisons in δ^13^C between recent and historical values [61]. However, the magnitude of the Suess Effect diminishes with increasing latitude in a non-linear manner, with step-effects in different oceanographic regions [53], [62–63]. Laysan albatross range from subtropical to subarctic waters and from mid-ocean to continental shelf, yet the foraging locations of our historically collected birds remain unknown. This lack of information inhibits our ability to reliably parameterize a calibration equation for the Suess Effect. Thus, we used δ^15^N but not δ^13^C for comparisons of mean values between historical and contemporary birds.

### Feather samples

We collected feather samples (1.2 mg, approximately 1 cm wide) composed of leeward feather barbs (not rachis) from the tip of the first feather and the base of the last feather of the annual molt sequence for the outer five primary feathers [64]. Samples from feather tips were collected 1–2 cm from the tip to reduce adverse effects on flight in live birds and allow others to still study feather wear in museum specimens.

Stable isotope values from feathers can be compared between seasons and years, but only if the sequence and timing of molt are known [65]. Flight feathers of albatross initiate growth at different but predictable times [64]. This provides the opportunity to compare stable isotope values of a single bird in different seasons. Feather sampling location (tip versus base) had an effect on δ^13^C (F_(1, 1018)_ = 120.317, p < 0.001), but not δ^15^N (F_(1, 1018)_ = 0.028, p = 0.87). Values for δ^13^C were higher at the base (-18.8 ‰ ± SD 0.8) than at the tip (-19.3‰ ± 0.8). Consequently, to test for effects of season, we did not compare tips to bases for δ^13^C, but we compared values from the tip of P6 and the base of P10 for δ^15^N. There was no effect on δ^15^N (F_(3, 145)_ = 1.385; p = 0.250) or δ^13^C (F_(3, 145)_ = 1.501; p = 0.217) based on which feather tip was sampled (P5, P6, P7, and P8). Therefore, feather identification (P5 –P8) was not included as a factor in subsequent analyses.

Albatross do not replace all their primary feathers each year [64]. Thus for samples collected in a single year, isotopic values can be compared between years. For Laysan albatross, the molt sequence of the outer primaries (P6-P10, where the highest number indicates the distalmost feather of the wing) varies annually between four distinct sequences. Molt initiates at feather P6, P7 or P8 and proceeds towards the wingtip, terminating at feather P10 [64], [66]. Feathers P8, P9 and P10 are always replaced. The inner primaries (P1-P5) initiate molt at P5 (or lower) and terminate at P1. The length of the flight feather molt season, ranging from May through October, is affected by the number of outer primaries replaced each year [32], [64], [67]. Based on this information, determination of the specific molt sequence of each bird sampled allowed us to estimate in which months each of our feather samples was grown (Table 1). Thus, although birds were sampled or collected across many different months of the year, we measured stable isotope values for every bird only for specific, bounded time frames, e.g., April-May or July-September.

**Table 1:** Feathers sampled and sampling location (within the feather), estimated months in which the sample was grown, estimated months of foraging represented by the sample, associated molt category (that determines feather to be sampled) and initiation and termination feather for each molt category.

Primary feather sampled	Feather sampling location	Months in which sample was grown^#^	Foraging months*	Molt category^#^	Feather that initiates molt in this molt category	Feather that terminates molt in this molt category
P5	tip	May	April	5	P6	
P6	tip	May	April	5, 53	P6	
P7	tip	May-June	April-May	4	P7	
P8	tip	June	May	3	P8	
P10	base	Aug-Oct	July-Sept	5		P10
P10	base	Oct	Sept	4, 3, 53		P10

^#^ Months of growth and molt category based on [64], [66–67].

* Prey consumption likely occurred about four weeks prior to the emergence above the sheath of the portion of feather sampled [70].

For all live birds sampled on the colony, feather wear was compared within each wing. This information was used to assign a molt category [68] and to determine whether feathers within a single wing presented one, or two, or three years of isotopic information. Because initiation of flight feather molt of the inner and outer series starts simultaneously when P6 is replaced [64], [66], it was also possible to use P5 to increase the sample size for inter-annual comparisons with P6, P7 and P8. Because P10 is molted every year, inter-year comparisons for the non-breeding (July-September) season were not possible. Because of limited access to specimens and lack of information on molt sequences, only P6 and P10 were sampled for fisheries-associated and historical birds. Thus, we were not able to examine inter-annual patterns for historical or fisheries-associated birds.

### Colony-based birds

Individuals from hatch years 1999 to 2003 were conspicuously color-banded as chicks by United States Fish and Wildlife Service (USFWS) personnel enabling us to detect their presence on the colony in 2007. We sampled 20 birds hatched in 2000 or 2001, aged 6 or 7 years, which are typical ages for courting prebreeders in January [69].

We collected feather samples from 111 randomly selected, individually banded, incubating birds of unknown age in long-term monitoring plots L7 and L10 from 5 to 24 January, 2007. Because egg laying begins in December, breeders that failed within the first three or four weeks of incubation, or skipped breeding entirely, were not sampled. USFWS personnel monitored the presence and breeding success of all banded birds. Monitoring data allowed us to divide our sampled birds into categories depending on 1) breeding status during the chick provisioning period of April-May (active versus failed), and 2) breeding quality (defined below). Breeders that failed at the incubation or hatching stage in 2006 (i.e., December 2005 –February 2006) and subsequently left the colony for the remainder of the breeding season were defined as failed breeders.

Laysan albatross show measurable trade-offs between sequential years in annual reproductive success, as demonstrated by the increased likelihood of skipped breeding and decreased likelihood of successful breeding in year 2 relative to higher breeding success in year 1 [29], a decrease in adult body condition (i.e., primary feather quality) in year 2 with increased breeding investment in year 1 [67], and the correlation between lower adult body condition (measured by accumulated worn feathers) and lower reproductive success [32]. Based on these quantifiable inter-annual trade-offs, we defined two categories for breeding quality. “Sequentially successful” breeders were birds that successfully fledged chicks in 2006 and 2007. “Sequentially unsuccessful” breeders were birds that fledged a chick in 2006 and then laid an egg but failed to fledge a chick in 2007. To control for the effect of breeding status (active versus failed) in April-May, we could only include in breeding quality categories birds that were successful, and thus “active” in 2006; and because all feather samples were collected during incubation in 2007, we could not include birds that were successful in 2006 but skipped breeding in 2007.

### Fisheries-associated birds

We obtained feather samples from bycaught birds from Alaskan and Hawaiian longline fisheries. All longline-salvaged albatross in this study were retrieved by fisheries observers, frozen, and returned to port. Birds in this study were caught in Hawaiian swordfish and tuna longline fisheries between January and April in 2002 (n = 2), 2005 (n = 13), and 2006 (n = 1); and were caught in Alaskan groundfish longline fisheries (n = 33) between November and July in every year from 2001 through 2006. Hawaiian-caught birds were sent to the Burke Museum at the University of Washington, Seattle, and Alaskan-caught birds were sent to the University of Alaska Museum, Fairbanks, Alaska, where feather samples were collected. For Alaskan-salvaged birds, 15 (collected January to May) were categorized as “after hatch year” (based on bursa size), nine (collected January to July) were categorized as “adult”, and six (collected November to December) were not identified to age class. There was no effect of known age class on δ^15^N (F_(1, 22)_ = 0.348; p = 0.53) or δ^13^C (F_(1, 22)_ = 0.698; p = 0.37).

### Historic birds

Feather samples were collected from specimens at the National Museum of Natural History, Smithsonian Institution, and the Burke Museum of Natural History. Specimens were evenly distributed between males and females, and were collected as adults in 1902 (3 birds), 1911 (3 birds), 1913 (3 birds) or 1923 (6 birds), February through May, from breeding colonies in the northwestern Hawaiian Islands (predominantly Laysan Island). There was no effect based on the year birds were collected on δ^15^N (F_(1, 13)_ = 2.334; p = 0.151) or δ^13^C (F_(1, 13)_ = 4.008; p = 0.066).

### Stable isotope analyses

Dry feather samples were placed in sealed vials and sent to the Colorado Plateau Stable Isotope Laboratory, Northern Arizona University, Flagstaff, Arizona, where they were solvent-washed with chloroform and methanol (2:1), ground, weighed, and analyzed for δ^13^C and δ^15^N values. Samples were run on a Thermo-Electron Delta V Advantage IRMS, configured through a Finnigan CONFLO III for automated continuous-flow analysis of δ^15^N and δ^13^C, using a Carlo Erba NC2100 elemental analyzer for combustion and separation of C and N. Based on 173 working standards of peach leaves, precision was 0.13‰ for δ^15^N and 0.05‰ for δ^13^C. Mean C/N mass ratio of 200 feather samples was 3.16 ± 0.07.

The isotopic value of assimilated prey is measurable in the isotopic value of a seabird’s growing feathers approximately four weeks after prey ingestion [70]. Thus, we subtracted one month from the estimated month of feather growth to estimate the month of foraging (Table 1).

### Statistical analysis

One-factor ANOVA with Bonferroni-corrected p-values for multiple comparisons were used to test for significant differences (α = 0.05) in mean stable isotope values within groups defined by hypothesized effects (e.g., breeding status, breeding quality, fishery, historic era). Two-way ANOVA with breeding category and year as factors was used to test for the effect of year on isotope values, and to test for inter-annual consistency in differences among breeding categories. Bartlett’s chi-square test was used to test for equality of variances. All statistical tests were performed in SYSTAT (ver. 11). Isotope values used in this study, identified by sampling category and individual bird, are accessible as Supporting Information (S1 Dataset).

## Results

**Fig 1:**
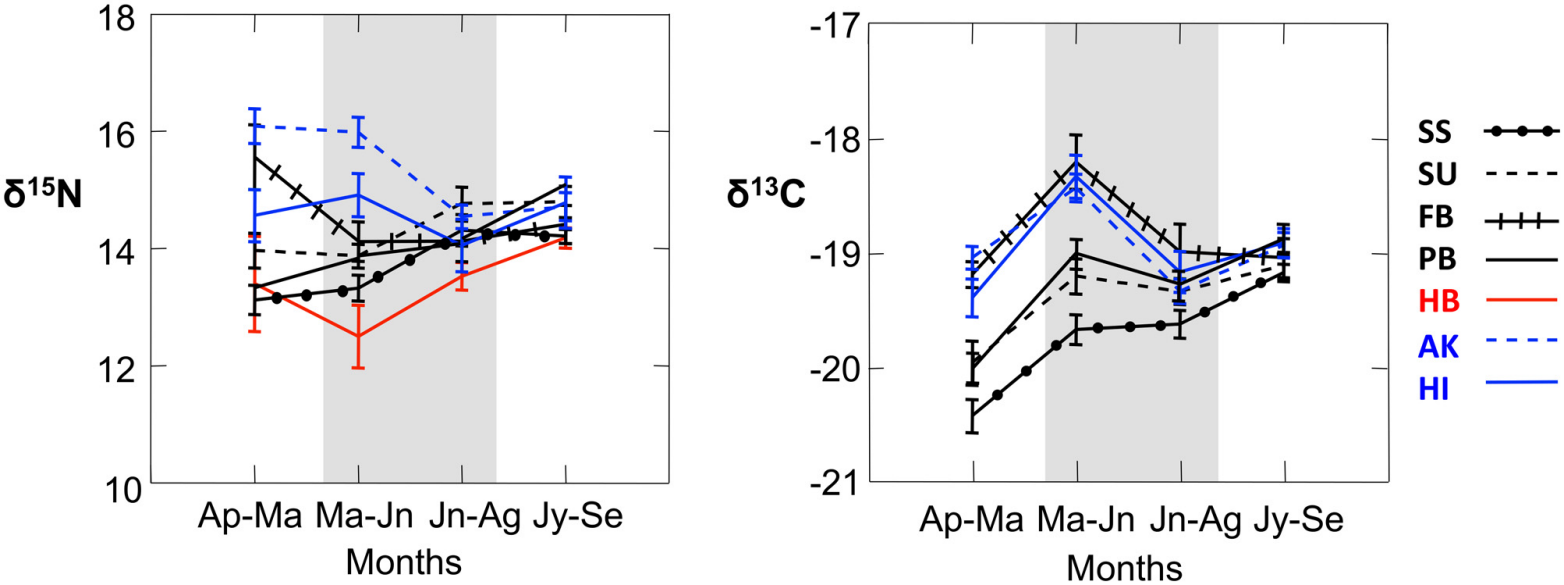
Nitrogen and carbon stable isotope values (mean± SE) from Laysan albatross foraging over four consecutive time periods as a function of sampling category. SS = sequentially successful breeders (fledged a chick in 2006 and 2007; feather samples from 2006); SU = sequentially unsuccessful breeders (fledged a chick in 2006, failed during incubation in 2007; feather samples from 2006); FB: failed breeders (failed during incubation in 2006, laid an egg in 2007; feather samples from 2006); PB: prebreeders (courting on Midway in January, 2007, feather samples from 2006), HB: historic birds, HI: Hawaiian fisheries-bycaught birds, and AK: Alaskan fisheries-bycaught birds. Data from sampling periods shaded in gray were not tested statistically because the breeding status of birds during these periods was uncertain due to variability of molt initiation dates.

For this study, we sampled feathers representative of foraging over four discreet time periods. We present information for all four time periods graphically for illustrative purposes of sequential change (Fig 1). However, we limited our statistical analysis to only two time periods: April-May representing the breeding season and specifically the mid-chick-rearing period, and July-September representing the non-breeding season when all birds were off the colony and, depending on their condition and status, were preparing for a return to the colony in November. It was only during these two extreme time periods that breeding status could be known with certainty due to the level of natural variability in molt initiation dates, and thus it was only during these two time periods that we could draw conclusions about the relationships between stable isotope values and breeding status.

### Breeding season–contemporary birds

Stable isotope values of Laysan albatross differed as a function of both breeding status and breeding quality. During the chick rearing season (April-May) of 2006, active breeders (AB; sequentially successful and sequentially unsuccessful birds combined) had significantly lower δ^15^N (AB vs FB − F_(1, 57)_ = 19.168; p < 0.001) and δ^13^C (F_(1, 57)_ = 23.306; p < 0.001) values than failed breeders (FB; Fig 1). Sequentially successful breeders (SS), which fledged chicks in consecutive years (2006, 2007), had significantly lower δ^15^N (SS vs SU − F_(1, 39)_ = 6.102; p = 0.020) but not lower δ^13^C (SS vs SU − F_(1, 39)_ = 3.413; p = 0.072) values relative to sequentially unsuccessful breeders (SU), which fledged in 2006 but laid an egg and then failed to fledge a chick in 2007 (Fig 1). For the chick rearing season (2006) prior to when they were observed courting on the colony in January (2007), prebreeders (PB) had stable isotope values similar to active breeders (AB vs PB vs FB- δ^15^N: F_(2, 76)_ = 13.211; p < 0.001; δ^13^C: F_(2, 76)_ = 11.766; p < 0.001; δ^15^N: p = 1.000; δ^13^C: p = 1.000; Fig 1), and significantly lower than those of failed breeders (δ^15^N: p < 0.001; δ^13^C: p = 0.003). Finally, while the variability in stable isotope values was comparable across colony-bound birds, it was significantly greater for failed breeders (Table 2). In sum, birds that left the colony early (FB) had a different mean and a greater variance in stable isotope values during the chick-rearing period compared to their colony-bound conspecifics (SS, SU, PB), suggesting different prey and/or different foraging locations. Among colony-bound birds, individuals with the highest success in sequential years (SS) appeared, on average, to forage on different prey and/or in a different location relative to less successful breeders (SU).

**Table 2:** Comparisons of variance between sampling categories during the breeding season (April-May) using Bartlett's chi-square test of variance equality.

	FB	AK	HI	PB	SU	SS
FB		0.151	0.278	**0.003**	**0.023**	**0.002**
		*2*.*059*	*1*.*177*	***9*.*002***	***5*.*145***	***10*.*026***
AK	0.397		0.924	**0.044**	0.248	**0.021**
	*0*.*718*		*0*.*009*	***4*.*064***	*1*.*333*	***5*.*343***
HI	0.219	0.565		0.063	0.282	**0.030**
	*1*.*509*	*0*.*332*		*3*.*447*	*1*.*158*	***4*.*704***
PB	**0.016**	**0.040**	0.253		0.390	0.617
	***5*.*844***	***4*.*207***	*1*.*306*		*0*.*738*	*0*.*251*
SU	**0.011**	**0.028**	0.212	0.916		0.198
	***6*.*387***	***4*.*835***	*1*.*555*	*0*.*011*		*1*.*657*
SS	0.446	**0.001**	0.651	0.110	0.089	
	*0*.*583*	***9*.*070***	*0*.*205*	*2*.*549*	*2*.*893*	

Above the diagonal refers to comparisons made for δ^15^N values. Below the diagonal refers to comparisons made for δ^13^C values. For each cell, the top number is the p-value and the bottom number (in italics) is the Chi^2^ value. P-values < 0.05 and associated chi-square values are in bold. FB = failed breeders. AK = Alaska fisheries-associated birds. HI = Hawaii fisheries-associated birds. PB = prebreeders. SU = sequentially unsuccessful breeders. SS = sequentially successful breeders. Sample size of each category reported in Table 3.

**Table 3:** Comparisons of variance between the breeding (April-May) and non-breeding (July–September) seasons, using Bartlett's chi-square test of variance equality.

		Nitrogen		Carbon	
Category	n	Chi^2^	p	Chi^2^	p
HB	15	**24.653**	**<0.001**	**5.420**	**0.020**
SS	16	**7.343**	**0.007**	**5.793**	**0.016**
SU	22	0.112	0.738	**4.489**	**0.034**
PB	20	0.027	0.870	2.535	0.111
FB	18	**4.332**	**0.037**	2.833	0.092
AK	33	1.550	0.213	0.494	0.482
HI	16	0.002	0.966	1.131	0.288

P-values < 0.05 and associated chi-square values are in bold. n = number of birds in each category. HB = historical breeders. SS = sequentially successful breeders. SU = sequentially unsuccessful breeders. PB = prebreeders. FB = failed breeders. AK = Alaska fisheries-associated birds. HI = Hawaii fisheries-associated birds.

**Fig 2:**
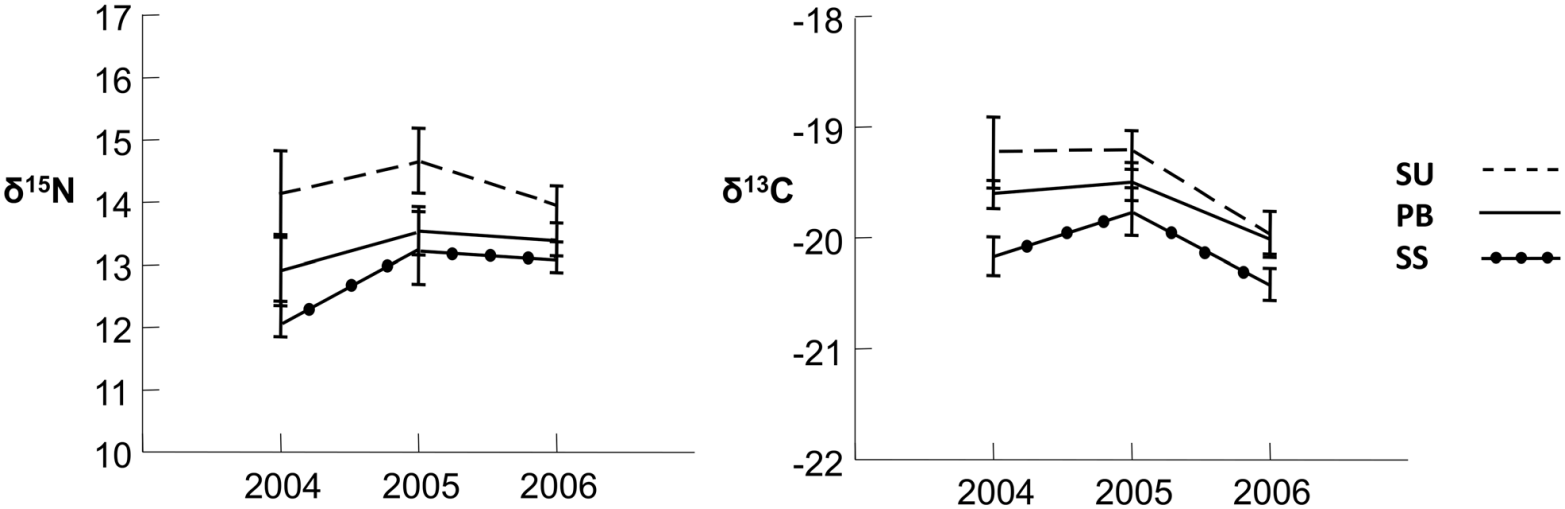
Mean (± SE) nitrogen and carbon stable isotope values for Laysan albatross foraging in April/May each year, as a function of multi-annual breeding success and breeding status. SS = sequentially successful breeders (fledged a chick in 2006 and 2007; n = 2, 12, 16 for 2004–2006, respectively); SU = sequentially unsuccessful breeders (fledged a chick in 2006, failed during incubation in 2007; n = 8, 19, 21); PB: prebreeders (courting on Midway in 2007 at ages 6 and 7 years old; n = 6, 12, 20).

Some of the birds found on the colony during the chick rearing season (AB, PB) retained flight feathers grown in sequential years, allowing examination of multi-year isotopic values (2005 and 2006; sample size for 2004 was too small to test statistically but is included in Fig 2). The relationships established in Fig 1 (2006) were generally consistent across years, despite an annual effect (Fig 2). Stable isotope values differed by year during chick-rearing only for δ^13^C, and by breeding category for both δ^15^N and δ^13^C (2-way ANOVA, δ^15^N: breeding category F_(1, 2, 96)_ = 4.948, p = 0.009, year F_(1, 2, 96)_ = 1.534, p = 0.218, with no interaction; δ^13^C: breeding category F_(1, 2, 96)_ = 4.016, p = 0.021, year F_(1, 2, 96)_ = 18.499, p < 0.001, with no interaction). Across both years, the δ^15^N and δ^13^C values of prebreeders did not differ from sequentially successful breeders (δ^15^N: p = 1.000; δ^13^C: p = 0.164); whereas prebreeders showed consistently lower δ^15^N values than sequentially unsuccessful breeders (δ^15^N: p = 0.018; δ^13^C: p = 0.525).

### Breeding season—historical comparisons

**Fig 3:**
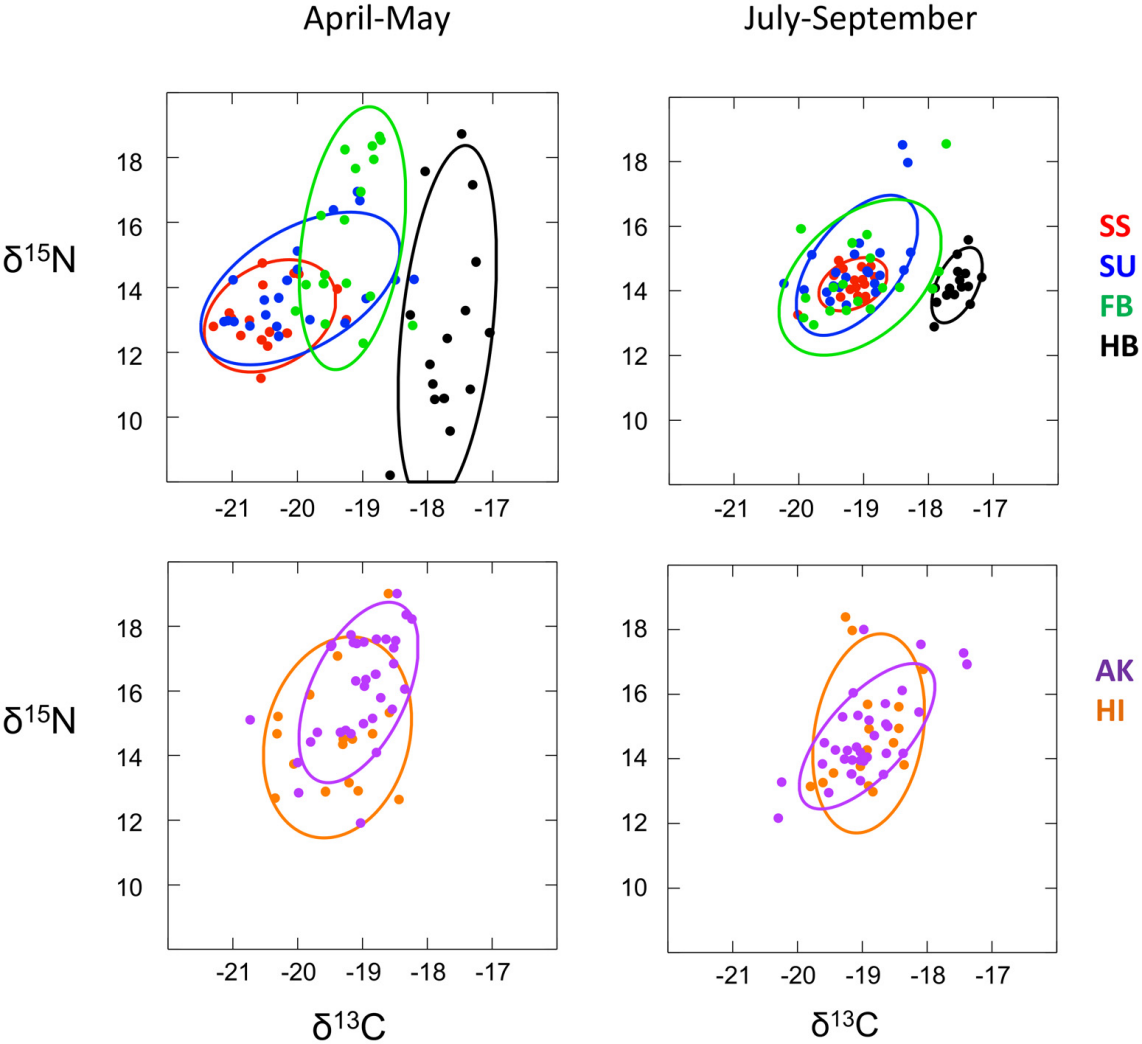
Nitrogen and carbon stable isotope values from individual Laysan albatross foraging during April-May (breeding season) and July-September (non-breeding season) as a function of sampling category. SS = sequentially successful breeders (fledged a chick in 2006 and 2007; feather samples from 2006); SU = sequentially unsuccessful breeders (fledged a chick in 2006, failed during incubation in 2007; feather samples from 2006); FB: failed breeders (failed during incubation in 2006, laid an egg in 2007; feather samples from 2006); HB: historic colony birds (δ^13^C not corrected for the Suess Effect); Hawaiian fisheries-bycaught birds; and Alaskan fisheries-bycaught birds. Ellipses encompass 75% of the estimated distribution for each category.

We assumed that our historic sample was originally collected on the breeding colonies without regard to breeding status. Therefore, we replicated that sample for contemporary birds; that is, we included δ^15^N values for P6 for all colony-sampled birds sampled in 2007, including birds not observed in 2006. Colony-sampled historic birds had lower mean δ^15^N values during chick-rearing relative to all colony-sampled contemporary birds (F_(1, 125)_ = 6.530, p = 0.012). Although colony-sampled contemporary and historic birds had similar upper δ^15^N values, lower values were different (contemporary: n = 112, mean = 14.4‰, SD = 3.1‰, range = 11.2 to 19.2‰; historic: n = 15, mean = 12.8‰, SD = 2.2‰, range = 8.2 to 18.7‰; Fig 3). Six out of 15 historic birds had δ^15^N values below 11.2‰, the lowest value observed for contemporary birds.

### Breeding season—comparison with fisheries-associated birds

During the chick-rearing period (April-May), birds salvaged from Alaskan fisheries (AK) had much higher δ^15^N values relative to birds salvaged from Hawaiian fisheries (HI; δ^15^N: F_(1, 47)_ = 8.734, p = 0.005) but only tended towards higher δ^13^C values (δ^13^C: F_(1, 47)_ = 3.975, p = 0.052; Fig 1) indicating that these birds may have been foraging on different prey and/or in different locations.

To examine possible relationships between breeding status and bycatch endangerment, we compared the isotope values of active breeders, failed breeders and fisheries-associated birds. Alaskan fisheries-associated birds (AB vs FB vs AK- δ^15^N: F_(2, 89)_ = 23.678; p < 0.001; δ^13^C: F_(2, 89)_ = 32.926; p < 0.001) had statistically similar stable isotope values to those of failed breeders (δ^15^N: p = 0.808; δ^13^C: p = 1.00) yet were considerably more enriched than active breeders (δ^15^N: p < 0.001; δ^13^C: p < 0.001; Fig 1). Hawaiian fisheries-associated birds fell in-between failed breeders and active breeders (which were distinct from each other), and were not statistically different from either (HI vs FB vs AB- δ^15^N: F_(2, 72)_ = 9.614; p < 0.001; δ^13^C: F_(2, 72)_ = 16.729; p < 0.001; failed breeders: δ^15^N: p = 0.235; δ^13^C: p = 0.235; active breeders: δ^15^N: p = 0.132; δ^13^C: p = 0.132; Fig 1). However, when active breeders were disarticulated by breeding quality, the higher isotope values of Hawaiian fisheries-associated birds were distinct from the values of sequentially successful breeders (HI vs SS vs SU- δ^15^N: F_(2, 50)_ = 4.514, p = 0.016; δ^13^C: F_(2, 50)_ = 8.385, p = 0.001; δ^15^N: p = 0.013, δ^13^C: p < 0.001), yet similar to values of sequentially unsuccessful breeders (δ^15^N: p = 0.580, δ^13^C: p = 0.061; Fig 1). During the breeding season, Alaskan fisheries birds (AK) also displayed greater variation in both δ^15^N and δ^13^C relative to all categories of colony-bound birds except δ^15^N of SU (Table 2; Fig 3). Hawaii fisheries birds (HI) were much less variable than Alaska fisheries birds, and relative to colony birds were only different than (greater than) the δ^15^N variability of sequentially successful breeders (SS; Table 2). Collectively, these results suggest that during the chick-rearing period at least some failed breeders may be foraging on similar prey types and/or in similar locations as birds caught in the Alaskan or Hawaiian longline fisheries. Furthermore, although some colony-bound birds may overlap in diet and/or location with Hawaiian fisheries-associated birds, the more successful breeders are less likely to do so.

Courting prebreeders had isotope means and variances that were distinctly lower than all Alaskan fisheries-associated birds combined (δ^15^N: F_(1, 51)_ = 40.906; p < 0.001; δ^13^C: F_(1, 51)_ = 24.555; p < 0.001; Fig 1, Table 2). Courting prebreeders also had distinctly lower isotope values than subadults (as a subcategory) caught by Alaskan fisheries (δ^15^N: F_(1, 33)_ = 13.453, p; < 0.001; δ^13^C: F_(1, 33)_ = 8.239; p = 0.004). Courting prebreeders had only somewhat lower isotope values than Hawaiian fisheries-associated birds (δ^15^N: F_(1, 34)_ = 6.225, p = 0.018; δ^13^C: F_(1, 34)_ = 5.547; p = 0.024; Fig 1) but similar variances (Table 2). In summary, prebreeders observed courting on the Midway colony in January appeared highly unlikely to associate with Alaskan fisheries during the chick-rearing season, and appeared to have only a low likelihood of association with Hawaiian fisheries.

### Non-breeding season

There was less isotopic variability during the non-breeding season (July-September, Table 4) relative to the chick-rearing season (April-May, Table 2), both within and among contemporary bird sampling categories (SS, SU, FB, PB, AK, and HI; δ^15^N: F_(6, 133)_ = 1.693, p = 0.127; δ^13^C: F_(5, 119)_ = 1.042, p = 0.396) due to a convergence towards intermittent values (Table 3, Figs 1 and 3). Note that historic birds were not included in the δ^13^C comparison of means because of the Suess Effect. Active breeders (AB) and prebreeders (PB) had higher δ^15^N values during the non-breeding season compared to the breeding season (April-May; AB: t = 5.682, df = 40, p < 0.001; PB: t = 5.343, df = 19, p < 0.001). By contrast, Alaska fisheries-associated birds had lower δ^15^N values (t = 4.169, df = 32, p < 0.001; Fig 1). Mean δ^15^N values for failed breeders (FB) and for Hawaiian fishery-associated birds (HI) did not differ between seasons (FB: t = 1.713, df = 17, p = 0.105; HI: t = 0.378, df = 15, p = 0.711; Fig 1).

**Table 4:** Comparisons of variance between sampling categories during the non-breeding season (July–September) using Bartlett's chi-square test of variance equality.

	HB	SS	SU	PB	AK	HI
HB		0.205	**0.015**	**0.034**	**0.004**	**0.001**
		*1*.*609*	***5*.*961***	***4*.*486***	***8*.*197***	***11*.*388***
SS	0.260		**<0.001**	**<0.001**	**<0.001**	**0.001**
	*1*.*271*		***13*.*289***	***11*.*151***	***16*.*442***	***20*.*078***
SU	**0.001**	**0.019**		0.708	0.647	0.175
	***10*.*823***	***5*.*551***		*0*.*140*	*0*.*210*	*1*.*837*
PB	**<0.001**	**0.009**	0.716		0.399	0.097
	***12*.*321***	***6*.*791***	*0*.*132*		*0*.*712*	*2*.*757*
AK	**<0.001**	**0.002**	0.413	0.686		0.287
	***15*.*270***	***9*.*294***	*0*.*671*	*0*.*163*		*1*.*135*
HI	**0.005**	0.067	0.645	0.436	0.230	
	***7*.*928***	*3*.*365*	*0*.*213*	*0*.*606*	*1*.*438*	

Above the diagonal refers to comparisons made for δ^15^N values. Below the diagonal refers to comparisons made for δ^13^C values. For each cell, the top number is the p-value and the bottom number (in italics) is the Chi^2^ value. P-values < 0.05 and associated chi-square values are in bold. HB = historical breeders. SS = sequentially successful breeders. SU = sequentially unsuccessful breeders. PB = prebreeders. AK = Alaska fisheries-associated birds. HI = Hawaii fisheries-associated birds. Sample size of each category reported in Table 3.

In addition to the seasonal shift in mean values, several categories, notably sequentially successful breeders, displayed significantly reduced variability in isotope values between the breeding and non-breeding seasons (Table 3). Of the remaining sampling categories, all of which were more variable relative to historic (HB) or sequentially successful breeders (SS) during the non-breeding season (Table 4), variances between seasons either did not differ from each other (PB, AK, HI) or differed in only one value (SU, FB; Table 4; Fig 3). These results suggest that during time of year when no birds were bound to the colony, diet and/or foraging location were more similar across the entire population.

## Discussion

Stable isotope analyses have demonstrated that foraging strategies of Laysan albatrosses are a complex function of season and breeding status, and possibly a function of experience and/or fitness, and that these factors likely affect the probability of association with fisheries. During the breeding season, birds with the highest, most consistent reproductive output displayed mean stable isotope values that were distinct from less productive conspecifics, and completely different from birds caught in Hawaiian or Alaskan longline fisheries (Fig 1). These differences were consistent within birds across years (Fig 2). As birds moved off the colony at the conclusion of the breeding season, the variable stable isotope values of birds during the breeding season, including birds sampled a century ago, coalesced into a more similar set of values during the non-breeding season (Fig 3).

### Foraging strategies as inferred from stable isotopes

Differences in foraging strategy may be caused by differences in prey composition of assimilated diet [46–51], foraging location [52–54], or both. On average, δ^13^C values are higher for nearshore, benthic food chains than for offshore, pelagic food chains, ranging from -17‰ for marine benthic algae to -22‰ for marine planktonic algae, due in part to differences in water turbulence and boundary layer thickness that cause carbon to be fractionated differently by pelagic and benthic primary producers [52]. Sea surface δ^13^C values are lowest in subtropical gyres [53]. Nitrogen also is fractionated differently by primary producers in higher productivity (nearshore, continental shelf) waters relative to lower productivity (offshore, mid-oceanic) waters [54].

In the eastern North Pacific Ocean, δ^15^N values of surface zooplankton are about 4‰ higher in shelf-associated waters compared to mid-oceanic waters [71–72]. In the western North Pacific Ocean, both δ^15^N and δ^13^C values are higher on the Japanese shelf and lower in the Oyashio current and the North Pacific Transition Zone [73]. Isotope values of seabirds follow these same geographic trends: higher among the neritic seabird guilds of the northern and eastern North Pacific Ocean [47–48] relative to the pelagic seabird guilds of the central North Pacific Ocean [41], [74–75]. In this study, Laysan albatross salvaged from Alaskan fisheries had considerably higher δ^15^N values and tended toward having higher δ^13^C values than Laysan albatross salvaged from Hawaiian fisheries, as predicted based on identifiable oceanographic gradients [52–54], [71–73].

The lower isotope values we observed for breeding season foraging by active breeders and courting prebreeders may be indicative of foraging in mid-oceanic waters on low- to mid-trophic level prey. Lower δ^15^N values have been correlated with higher body condition in some marine birds [76]. In contrast, the higher mean δ^15^N and δ^13^C values of failed breeders may be indicative of foraging on or near the continental shelf where the Alaskan longline fisheries are concentrated, and/or on higher-trophic level prey in mid-oceanic waters, including fisheries-associated food such as tuna or swordfish. Stable isotope studies in the South Atlantic of ship-following seabirds have attributed enriched δ^15^N values of blood to the consumption of fisheries-associated food [77–78]. We did not separate the effect of foraging location from the effect of prey type on stable isotope values, so we can not conclude with certainty from this study alone that elevated δ^15^N or δ^13^C among fisheries-associated birds were the direct result of consuming fisheries-associated food.

Sequentially successful breeders had low isotopic variability relative to birds of lesser breeding success, and relative to fisheries-associated birds (Tables 2 and 3, Fig 3). Reduced foraging heterogeneity is associated with higher reproductive success [25]. Natural selection acting on younger and less experienced breeders can effectively reduce foraging heterogeneity with selection intensifying during years of low food availability [24–26], [79]. The result is that older or more experienced breeders exhibit narrower foraging breadth. Our results follow predicted patterns and suggest that our sample of sequentially successful breeders may have been biased towards experienced breeders. For the first time for North Pacific albatrosses, foraging breadth has been linked to fitness. Given the potential importance of this link to population modeling and management, further studies with larger sample sizes and more years of breeding data are warranted.

During the breeding season, the lower mean values of colony-bound birds, especially sequentially successful breeders, were notably similar to the lower mean values of historic birds. During the non-breeding season, historic birds and sequentially successful breeders had notably small variances, relative to all other categories, around similar means (Table 4, Fig 3). Taken together, these similarities suggest that despite new foraging opportunities or constraints that have developed over the last century (e.g., climate change and the advent of industrial fishing), contemporary birds with the highest reproductive success may retain more than any other group the foraging strategies of their forebears.

During the breeding season we observed inter-annual consistency in mean isotope values among sequentially successful breeders, sequentially unsuccessful breeders, and courting prebreeders, even as we observed an effect of year (Fig 2). One interpretation is that individual personality and learning [19–20], [73] [74] affect Laysan albatross foraging patterns at a young age, persisting even during measurable inter-annual variation in foraging opportunities [14]. The degree to which these persistent foraging differences influence reproductive output remains unknown, although the pattern is intriguing.

### Associations with fisheries

Our results suggest that adult Laysan albatross caught in Alaskan longline fisheries were more likely to be failed breeders than colony-bound birds such as active breeders or courting prebreeders (Fig 1). Although the majority of fishing effort in the sablefish, turbot and cod longline fisheries in Alaska occurs from September through May, bycatch of Laysan albatross peaks from April through July (1995–2001) [44], a time of declining colony attendance because of breeding failures [69]. Tracking studies indicate that active breeders spend only a minority of time in Alaskan waters during the breeding season [16], [37], likely because reproductive success is inversely correlated with maximum foraging distance [14], [17], [22].

The majority of our Alaskan fisheries bycaught birds were prebreeders (15 out of 24 birds, categorized by bursa size). Actively courting, six and seven year old prebreeders observed on the colony in January, had demonstrably different isotope values than Alaskan fisheries-associated prebreeders (of unknown ages). Prebreeders three years and older are found on Midway from March through May, arriving progressively earlier each subsequent year [69]. Adult breeders arrive on the colony in late October or November [69]. Good body condition is necessary before birds begin actively breeding [33], [80]. Therefore, acquisition of colony-centered foraging skills, a set of knowledge and abilities that may increase year by year as prebreeders age and learn [19–20], [81], [82], are probably necessary for successful breeding. Whether or not to spend time with fishing vessels [7] must in part be a learned behavior, possibly shaped at a young age. Based on the results of this study we suggest that for Laysan albatross, the status “prebreeder” could be divisible as a function of colony association.

Despite fishing effort throughout the year, bycatch of Laysan albatross in Hawaiian tuna and swordfish fisheries is greatest from January to May, months of high attendance on the breeding colonies, (2000 to 2006, north of 23°N latitude where most Laysan albatross are caught; Pacific Islands Regional Office, NOAA, unpublished data). This suggests that the proximity of the Hawaiian fisheries to active breeding colonies may affect the likelihood that Laysan albatross will associate with these fisheries. Based on stable isotope results, we can not rule out that active breeders or prebreeders from Midway might associate with these fisheries in April-May. However, our results suggest that especially the higher quality breeders, and possibly the courting prebreeders observed on the colony in January, have lower probabilities than other colony-bound birds of associating with Hawaiian tuna and swordfish fisheries (Fig 1).

The distant water tuna fisheries of Japan and Taiwan operate in the western North Pacific within core Laysan albatross foraging areas of the North Pacific Transition Zone (NPTZ) [1], [45]. Laysan albatross comprise 60% of all seabirds caught in these longline tuna fisheries, and bycatch rates are higher per unit fishing effort than in Hawaiian or Alaskan longline fisheries, in part because seabird bycatch mitigation measures are not mandatory [45]. Tracking studies can assess probabilities of geographic overlap. However, feather stable isotope values from birds caught in these fisheries could provide greater clarity about the degree to which demographic, seasonal or historic factors may affect the likelihood of hooking and drowning, information that could facilitate improved bycatch mitigation efforts.

The high variability of isotope values among fisheries-associated birds (Tables 2 and 3, Fig 3) based on feather samples grown across multiple years, suggests that birds known to associate with Alaskan or Hawaiian fisheries do not, as a group, appear to specialize on association with fisheries, but instead exploit a diversity of foraging opportunities.

### Historic and seasonal patterns

When all contemporary colony birds were combined and only values from the tip of P6 were included, δ^15^N values during the breeding season were lower, on average, a century ago, and the range of values was much greater than the range of values observed today. Both contemporary and historic sampling categories included birds of unidentified age and reproductive output but each likely included a mix of active and failed breeders (feather samples represent foraging one, two or three years prior to collection on the colony), which could explain a significant proportion of the variance observed for the historic sample (Fig 3).

For historic birds, the highest values of δ^15^N were not different than values observed for contemporary Alaska fisheries-associated birds (Fig 3). The similarity of high-ranging δ^15^N values before and after the advent of industrial longline fishing does not lend support to the assumption that Alaskan fishery-associated birds today have enriched isotope values primarily because of consumption of fisheries-associated food. Instead, the similarity of high-ranging values before and after the advent of industrial fishing in Alaska suggests that Laysan albatross foraged a century ago, just as they do today, on the continental shelf.

In contrast, the very low δ^15^N values observed for historic birds but not for contemporary birds suggest Laysan albatross may once have fed more commonly on low-trophic level prey in low productivity waters such as the subtropical gyre. Today, the subtropical gyre is expanding, causing the core foraging grounds of Laysan albatross in the NPTZ to move a predicted 600 km further north by 2100 [83–84]. The historic presence but contemporary absence of very low δ^15^N values in our study (e.g., 8.2‰) is worth investigating further with a larger sample size of historic birds to better understand the dynamics in which Laysan albatross might have foraged in lower productivity waters on lower trophic level prey.

The decrease from spring to late-summer in high-ranging δ^15^N values for both historic and contemporary birds suggests that environmental factors drive a shift from nearshore to offshore foraging, and/or a shift in availability from high to mid or low trophic level prey. Environmental factors that could drive these seasonal changes in foraging location and/or prey composition include large-scale oceanographic processes [85–86] that affect the timing, location, and growth rates of a range of species found in the northern NPTZ [87], including species known to be prey of Laysan albatross [41].

### Future directions

Our stable isotope results suggest that foraging strategies of Laysan albatross differ with season, and between demographic groups. Future research should determine how much the variation in foraging strategies is due to differences in foraging location, and how much is due to differences in prey type, including the proportion of fisheries-associated food that is actually consumed when albatross and fisheries overlap spatially. This latter measure may be a better indicator of the degree of direct association of Laysan albatross with fishing vessels, because relative proximity alone does not necessarily lead to ship following [7].

For far-ranging seabirds, methods of direct diet sampling (stomach contents or regurgitated pellets) are generally inadequate for making demographic or seasonal distinctions in prey composition due to the inability to reliably access representative samples in different demographic categories across seasons. For example, stomach contents for Laysan albatross have been obtained primarily from dead fisheries bycaught birds [41], whose diet choices, as suggested by this stable isotope study, may not be representative of Laysan albatross in all behavioral categories. Regurgitated pellets are limited to colony-bound chicks and adults, usually later in the breeding season and without reference to demographic differences, and are inherently biased towards prey species with indigestible parts such as squid [38–40].

Tracking studies are ideally suited for assessing geographic overlap of North Pacific albatrosses with regional fisheries [5], [88], as well as for measuring foraging association with remotely sensed oceanographic features, such as sea surface temperature, hydrographic fronts or wind [14], [21], [37], [78], [89]. However, tracking studies alone can not determine which prey are consumed when an individual travels to different parts of its foraging range.

Stable isotope studies from feathers retain a unique methodological niche by providing standardized measures across the full range of bird sampling categories with results specific to season and year, assuming the seasonal and annual timing of feather growth patterns are known with certainty [51], [90]. The next step for Laysan albatross stable isotope research would be to separate the effects of foraging location from the effects of prey type. The effect of foraging location can be measured by analyzing isotopic values of feathers of known seasonality collected from tagged individuals that have seasonally identifiable foraging ranges [59]. The effect of prey composition can be measured by first measuring stable isotope values for a range of location-specific prey (including fisheries-associated food), then develop location-specific stable isotope mixing models [77–78]. These stable isotope research efforts would facilitate modeling of the demographic consequences of both fisheries-associated mortality (e.g., [91]), and changing foraging dynamics due to climate change [78].

## Supporting Information

S1 Dataset Sampling category, season, year, and source information for each feather sample. (XLSX)
